# Effects of *Capsicum annuum* supplementation on the components of metabolic syndrome: a systematic review and meta-analysis

**DOI:** 10.1038/s41598-020-77983-2

**Published:** 2020-12-01

**Authors:** Hwan-Hee Jang, Jounghee Lee, Sung-Hyen Lee, Young-Min Lee

**Affiliations:** 1 Functional Food Division, National Institute of Agricultural Sciences, Rural Development Administration, Wanju, 55365 South Korea; 2 Department of Food and Nutrition, Kunsan National University, Gunsan, 54150 South Korea; 3 Division of Applied Food System, Major of Food and Nutrition, Seoul Women’s University, Seoul, 01797 South Korea

**Keywords:** Diseases, Medical research

## Abstract

Metabolic syndrome (MetS) has increasingly gained importance as the main risk factor for cardiovascular diseases and type II diabetes mellitus. Various natural compounds derived from plants are associated with beneficial effects on the incidence and progression of MetS. This study aimed to evaluate the effects of *Capsicum annuum* on factors related to MetS by assessing randomized controlled trials (written in English). We searched the online databases of PubMed, Embase, Google scholar, and Cochrane Library up to April 2020. ‘Patient/Population, Intervention, Comparison and Outcomes’ format was used to determine whether intervention with *C. annuum* supplementation compared with placebo supplementation had any effect on the components of MetS among participants. We considered standardized mean differences (SMD) with 95% confidence intervals (CI) as effect size measures using random-effects model. Analysis of the included 11 studies (n = 609) showed that *C. annuum* supplementation had significant effect on low density lipoprotein-cholesterol [SMD = − 0.39; 95% CI − 0.72, − 0.07; *P* = 0.02; prediction interval, − 1.28 to 0.50] and marginally significant effect on body weight [SMD = − 0.19; 95% CI − 0.40, 0.03; *P* = 0.09]. However, larger and well-designed clinical trials are needed to investigate the effects of *C. annuum* on MetS.

## Introduction

Metabolic syndrome (MetS), also known as syndrome X, is a clinical condition characterized by abdominal obesity, insulin resistance, hypertension, and dyslipidemia^1^. MetS has increasingly gained importance as the main risk factor for cardiovascular disease (CVD) and type II diabetes mellitus (T2D), which can lead to mortality^2^. Risk factors for CVD and T2D include hypertension, dyslipidemia, and high fasting glucose, which, in fact, characterize MetS^3–5^. Since the prevalence of the MetS is among a quarter of the global population, it has become a problem not only in the Western world but also in developing countries^6^.

MetS has been defined by various healthcare organizations, with minor differences in the definitions^7^. Four components, namely, obesity, hypertension, hyperglycemia, and dyslipidemia, are commonly used to describe this condition^3^. However, these factors could be improved to prevent MetS; this can be done by adopting healthier lifestyle habits such as dietary modification. Particularly, various natural compounds derived from plants are associated with beneficial effects on the incidence and progression of MetS^8^.

*Capsicum annuum* is a pungent spice, which is also known as red pepper or chili pepper. The capsaicinoid in the spice is responsible for its pungency. The main capsaicinoid is capsaicin, which is characterized by its chemopreventive, antioxidant, anti-inflammatory, hypolipidemic, thermogenic, and weight-reducing effects^9^. Recently, a non-pungent compound, capsinoid (e.g., capsiate, dihyrocapsiate), was discovered. Though few systematic reviews were conducted to provide an overview of the effects of *C. annuum* on factors related to MetS^10,11^, no meta-analysis (the quantitative summary of different or conflicting results) has been conducted in this regard. Therefore, the aim of this study was to evaluate the effects of *C. annuum* on factors related to MetS by focusing on relevant and related randomized controlled trials (RCTs). We determined whether an intervention with *C. annuum* supplementation compared with placebo supplementation had any effect on the components of MetS among the participants.

## Results

### Identification and selection of studies

**Figure 1:**
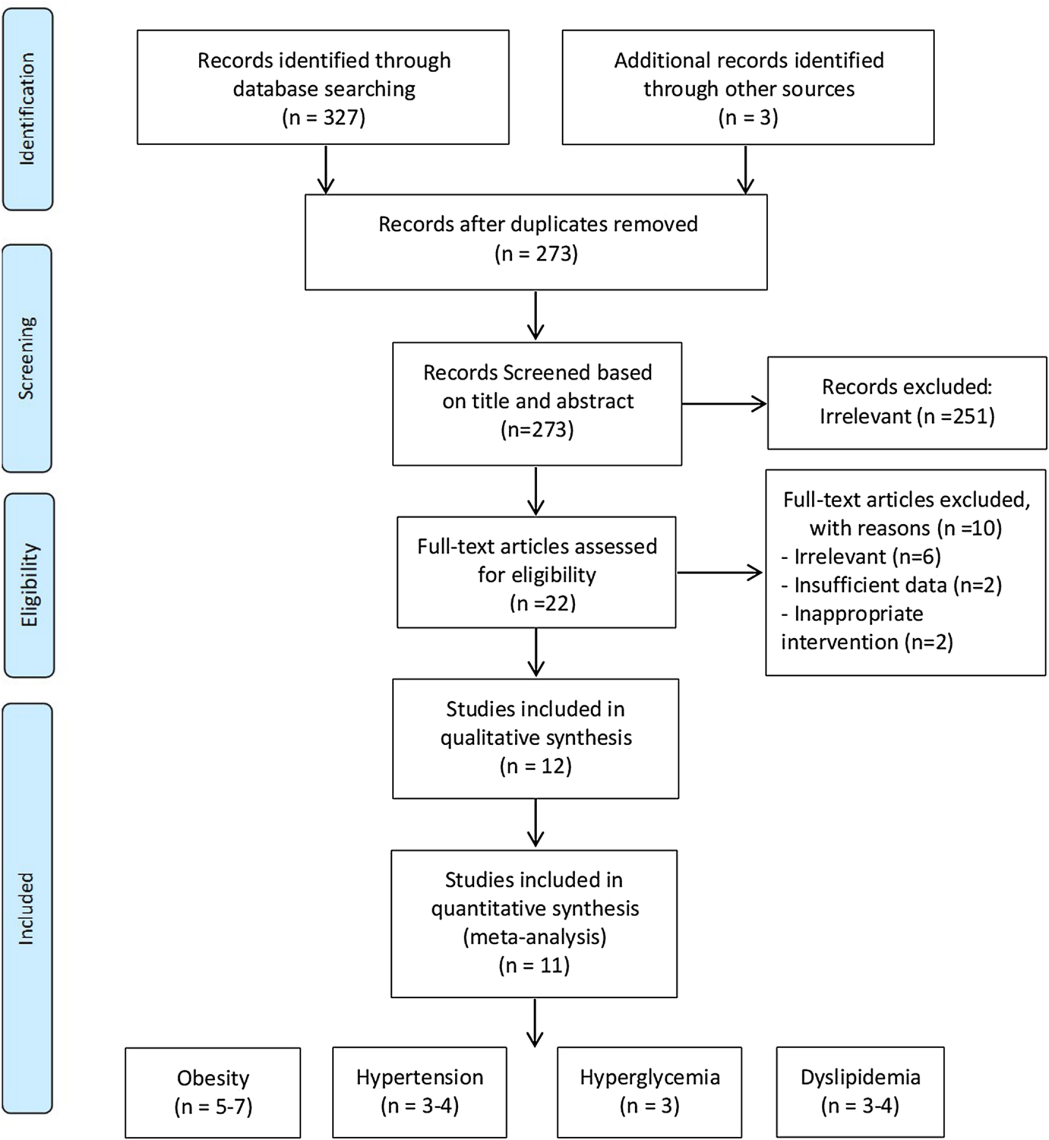
Preferred reporting items for systematic reviews and meta-analyses flowchart.

A total of 327 studies were identified from database search and three additional articles were identified from manual search. There were 273 articles after removing duplicate results; 251 trials were subsequently excluded by two authors based on their titles and abstracts. The remaining 22 articles were reviewed for dual full-text screening. After the review, a total of 6 trials were excluded. Thereafter, two papers were excluded due to insufficient data presentation^12,13^ and two more were excluded due to inappropriate interventions^14,15^, leaving a total of 12 studies, which were included in the systematic review. In meta-analysis, one study that has patients as subjects was excluded^16^. The whole selection process is presented in the Preferred Reporting Items for Systematic Reviews and Meta-Analyses flow diagram (Fig. 1).

### Description of included studies

A total of 12 studies were included in the systematic review. The characteristics and findings of the included studies are summarized in Table 1. Most studies included both sexes, except for three studies that included only females^16–18^ and one study that included only males^19^. All studies were conducted on adults aged over 18 years; one study was conducted on middle-aged people and elderly (over 50 years)^20^. Out of the 12 studies, five were conducted in the United States^18,19,21–23^, three in Korea^17,24,25^, two in Japan^20,26^, one in Australia^27^, and one in China^16^. Ten of the studies were parallel-arm studies^16,17,19–26^ whereas the other two were crossover studies^18,27^. Ten studies were conducted on individuals with body mass index (BMI) above 25 kg/m^2^^16–19,21–25,27^, and two studies were conducted on healthy individuals with BMI below 25 kg/m^2^^20,26^. Six studies focused on intervention with purified capsaicinoids or capsinoids capsules^19–23,26^, three studies were on pepper powder or juice supplementation^16,18,27^, and another three were on fermented red pepper paste supplementation^17,24,25^. The duration of the interventions in the included studies varied from 4 to 12 weeks.

**Table 1:** Characteristics and findings of the studies included in the systematic review.

Study (Ref)	Design	Place	Subjects (% of women)	Age (year)	BMI	Intervention			Control			Duration	Outcome
						n	Type	Dose/day	n	Type	Dose/day		
Ahuja et al. 2007^27^	Cross-over	Australia	36 (61)	46.0	26.4	36	Freshly chopped chilli	30 g	36	Chilli-free	Without any chilli	4 weeks	BMI, BF, BP glucose, TG, TC, HDL-C, LDL-C
Cha et al. 2013^24^	Parallel-arm	Korea	60 (88)	42.6	27.1	30	Fermented red pepper paste (FRPP) pills	32 g (FRPP 11.9 g)	30	Placebo pills	32 g	12 weeks	BMI, BF, BW, BP, glucose, TG, TC, HDL-C, LDL-C
Galgani and Ravussin 2010^19^	Parallel-arm	USA	78 (0)	36.7	29.4	Each 25	Dihydrocapsiate (DCT) capsules	3 or 9 mg	28	Placebo capsules	0 mg	4 weeks	BF
Kim et al. 2010^17^	Parallel-arm	Korea	28 (100)	18–60	26.7	14	FRPP pills	32 g (FRPP 11.9 g)	14	Placebo pills	0 g	12 weeks	BMI, TC, LDL-C
Lee et al. 2010^21^	Parallel-arm	USA	46 (NI)	51.6	30.9	15 or 16	DCT capsules	DCT 3 mg or 9 mg	15	Placebo capsules	0 mg	4 weeks	BW
Lim et al. 2015^25^	Parallel-arm	Korea	30 (57)	42.0	26.9	15	FRPP pills	34.5 g	15	Placebo pills	34.5 g	12 weeks	TG, HDL-C, TC, LDL-C
Nieman et al. 2012^18^	Cross-over	USA	31(100)	57.7	> 27	31	Red pepper capsules	1 g	31	Placebo capsules	White rice flour	4 weeks	BF, BW, BP, glucose
Nirengi et al. 2016^26^	Parallel-arm	Japan	20 (50)	20.8	21.7	10	Capsinoids capsules	9 mg capsinoid	10	Placebo capsules	0 mg	8 weeks	BMI, BW, BP
Rogers et al. 2018^22^	Parallel-arm	USA	77 (61)	29.6	27.4	27 or 22	Capsimax (capsules)	Capsaicinoid 2 mg or 4 mg	28	Placebo	Corn starch	12 weeks	BF
Snitker et al. 2009^23^	parallel-arm	USA	67 (52)	42.0	30.5	31	Capsinoids capsule	6 mg	36	Placebo capsule	0 mg	12 weeks	BW
Yokoyama et al. 2020 ^20^	Parallel-arm	Japan	69 (75)	74.1 (> 50)	23.4	36	Capsinoids capsule	9 mg capsinoids	33	Placebo capsule	0 mg	12 weeks	BMI, BW
Yuan et al. 2016^16^	Parallel-arm	China	44 (100)	30.5	27.1	20	Chili powder	1.25 g (5 mg/day of capsaicin)	22	Chili powder	1.25 g/0 mg of capsaicin	4 weeks	BMI, BW, BP, Glucose, TG, TC, LDL-C

*NI* no information, *BMI* body mass index, *BW* body weight, *BF* body fat, *BP* blood pressure, *TG* triacylglycerol, *TC* total-cholesterol, *HDL-C* high density lipoprotein-cholesterol, *LDL-C* low density lipoprotein-cholesterol.

### Potential sources of bias

**Figure 2:**
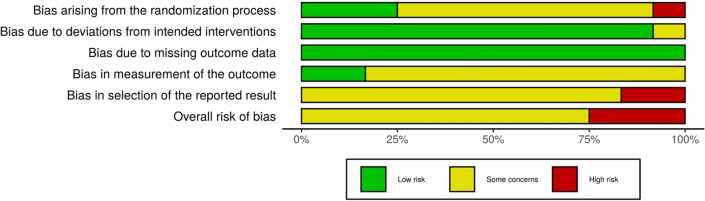
Summary plot of risk of bias.

The Risk of Bias (ROB) assessments for individual studies are presented in Fig. 2. Three out of the 12 included studies had a high ROB, whereas the remaining nine studies had some ROB concerns. Bias arising from the randomization process was low in three studies^16,20,25^, eight studies had some ROB concerns^17,19,21–24,26,27^, and one study had a high ROB^18^ because the only information in this article was a sentence stating that the study was randomized. Bias due to deviations from the intended intervention were low in all studies except for one^17^, in which allocation concealment was not reported. Bias due to missing outcome data was low in all the 12 studies. Two studies^16,20^ were rated with a low ROB in the measurement of outcomes, whereas 10 articles did not indicate whether the outcome assessors were aware of the intervention the study participants underwent. Two studies, in which only subgroup results were presented without the overall results^17^ and the results of follow-up were not reported^25^, were rated with a high ROB in the selection of the reported result.

### The effects of *C*. *annuum* supplementation on obesity

**Figure 3:**
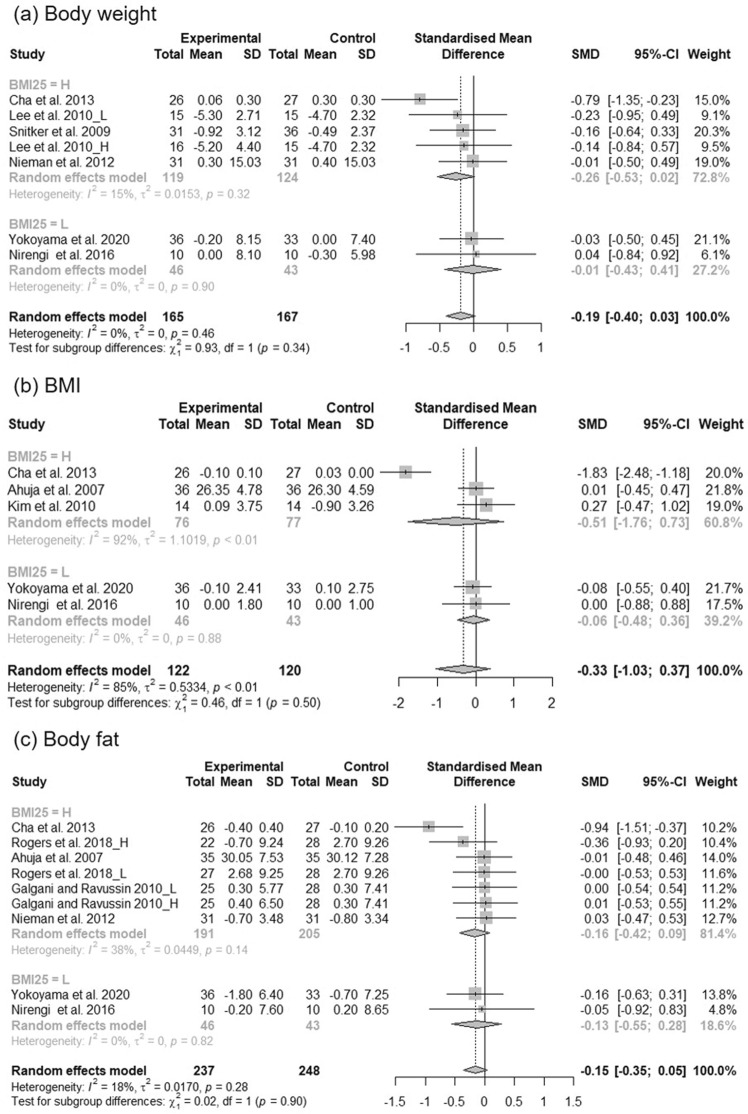
Forest plot of the changes in the standardized mean differences (with 95% confidence intervals) of body weight (**a**), BMI (**b**), and body fat (**c**) in participants treated with *C*. *annuum* compared with controls.

The forest plot for overall random effects of *C. annuum* supplementation on anthropometric parameters in participants is shown in Fig. 3. Studies were grouped into those involving participants with low (< 25 kg/m^2^) and high (≥ 25 kg/m^2^) BMI. The meta-analysis on body weight involved five results from four studies that included participants with high BMI versus two studies that included participants with low BMI. The overall pooled statistics showed that the body weight of participants (seven results from six studies) were marginally reduced [standardized mean differences (SMD) = − 0.19; 95% confidence intervals (CI) − 0.40, 0.03; *P* = 0.09]. There was no statistically significant heterogeneity between the studies in the analysis of body weight [I^2^ = 0% and *P* = 0.46]. The effect of *C. annuum* was not significantly different between subgroups according to BMI. Five effect sizes from five studies on BMI and nine effect sizes from seven studies on body fat were included in the analysis of the impact of *C. annuum* (Fig. 3). The SMD of the overall pooled BMI and body fat was − 0.33 [95% CI − 1.03, 0.37; *P* = 0.36] and − 0.15 [95% CI − 0.35, 0.05; *P* = 0.13], respectively; the results did not decrease significantly. However, considerable heterogeneity was detected in the analysis of BMI [I^2^ = 85% and *P* < 0.01]. Sensitivity analyses of the effect of *C. annuum* supplementation on BMI showed that removal of any study did not alter the significance of the pooled effect size (Supplementary Fig. S1). However, removal of some studies changed the significance of the heterogeneity. Heterogeneity between studies regarding the pooled effect size of BMI reduced to 0% when the study of Cha et al.^24^ was removed. Each study’s contribution to the heterogeneity is presented by the Baujat plot in Supplementary Fig. S1.

### The effects of *C*. *annuum* supplementation on hypertension

**Figure 4:**
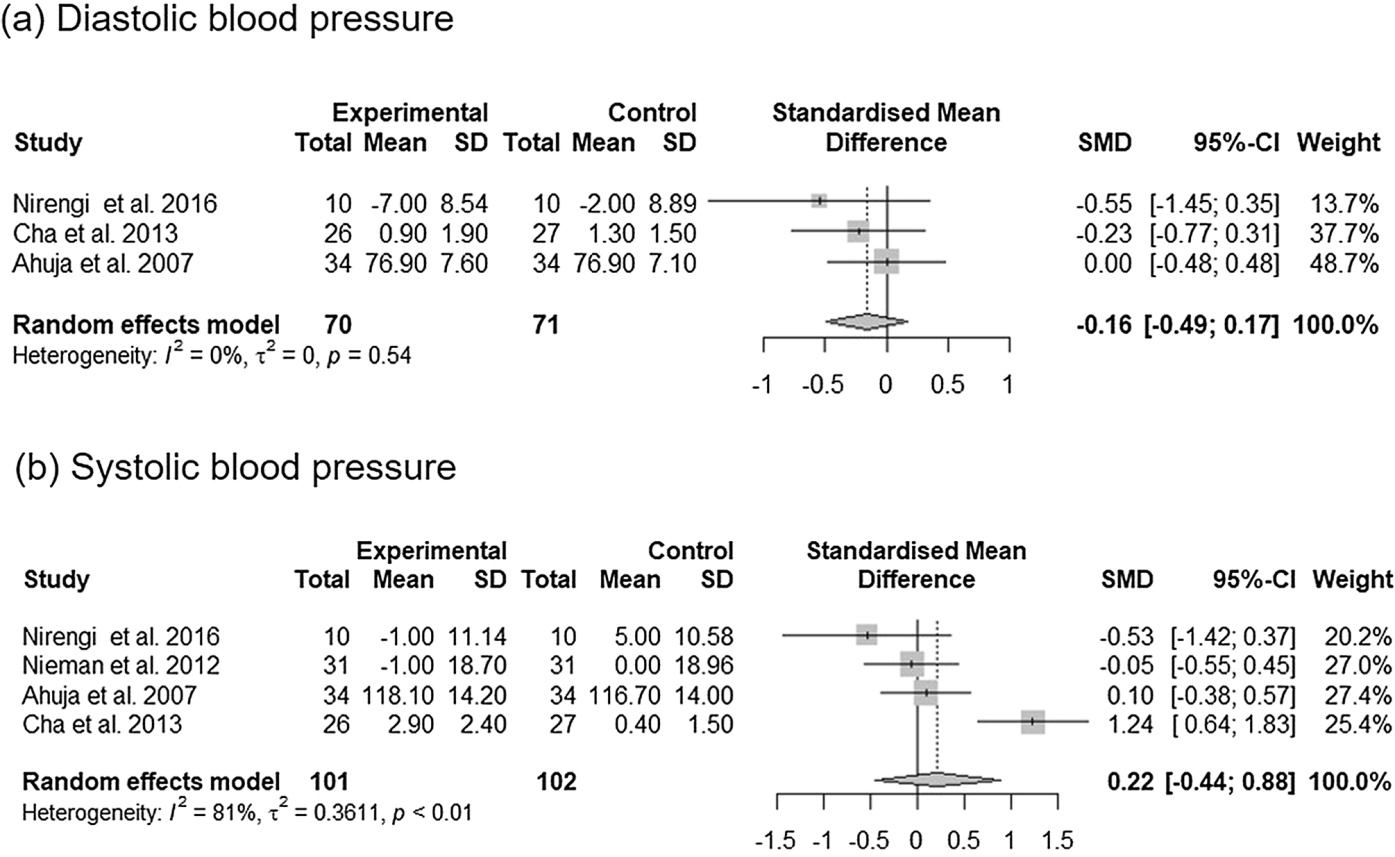
Forest plot of the changes in the standardized mean differences (with 95% confidence intervals) of diastolic blood pressure (**a**) and systolic blood pressure (**b**) in participants treated with *C*. *annuum* compared with controls.

Overall, three effect sizes from three studies on diastolic blood pressure (DBP) and four effect sizes from four studies on systolic blood pressure (SBP) were included in the analysis of the impact of *C. annuum* (Fig. 4). *C. annum* supplementation had no significant effect on DBP [SMD = − 0.16; 95% CI − 0.49, 0.17; *P* = 0.34] and no significant between-study heterogeneity was observed in the analysis [I^2^ = 0% and *P* = 0.54]. Although *C. annum* supplementation had no significant effect on SBP [SMD = 0.22; 95% CI − 0.44, 0.88; *P* = 0.52], a significant heterogeneity was observed in the analysis of SBP [I^2^ = 81% and *P* < 0.01]. Sensitivity analyses of the effect of *C. annuum* supplementation on SBP showed that removing any study did not alter the significance of the pooled effect size but changed the heterogeneity to 0% when the study of Cha et al.^24^ was removed (Supplementary Fig. S2).

### The effects of *C*. *annuum* supplementation on hyperglycemia

**Figure 5:**
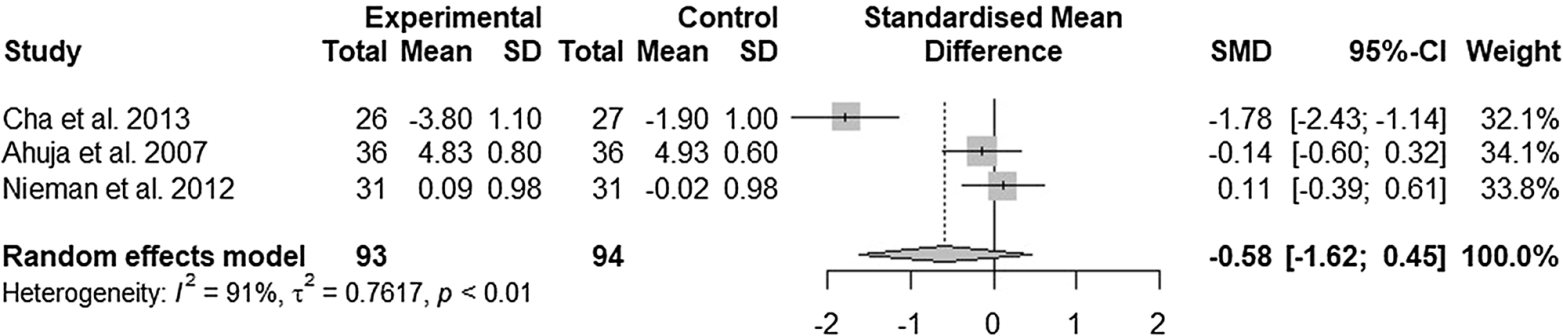
Forest plot of changes in the standardized mean differences (with 95% confidence intervals) of blood glucose levels in participants treated with *C*. *annuum* compared with controls.

Three studies investigated the outcomes and impact *C. annuum* supplementation on glucose levels (Fig. 5). *C. annuum* supplementation had no significant effect on glucose levels [SMD = − 0.58; 95% CI − 1.62, 0.45; *P* = 0.27] but the analysis showed considerable heterogeneity [I^2^ = 91% and *P* < 0.01] between studies. Sensitivity analyses of the effect of *C. annuum* supplementation glucose levels showed that removal of any study did not alter the significance of the pooled effect size but lowered the heterogeneity to 0% when the study of Cha et al.^24^ was removed (Supplementary Fig. S3).

### The effects of *C*. *annuum* supplementation on dyslipidemia

**Figure 6:**
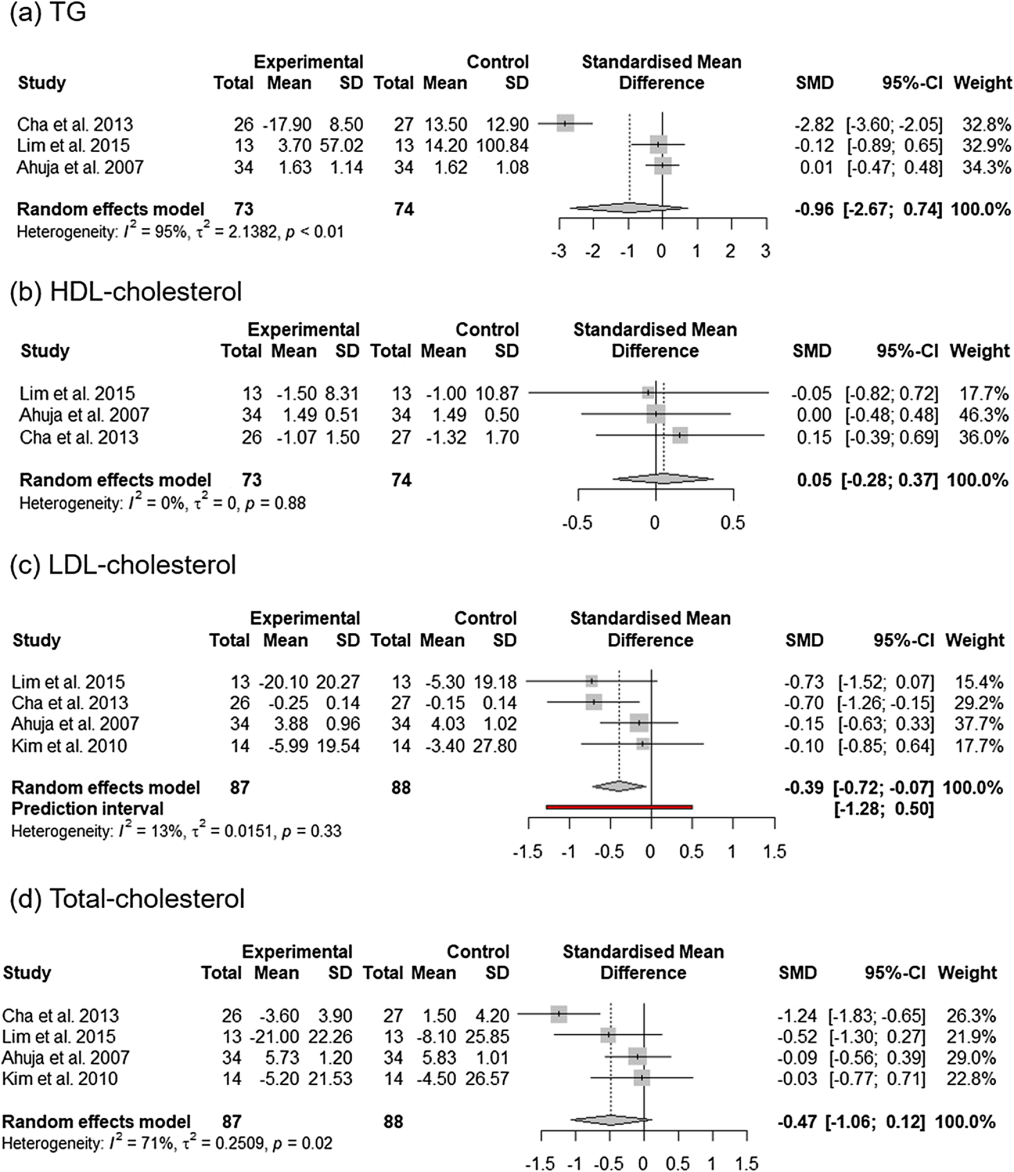
Forest plot of changes in the standardized mean differences (with 95% confidence intervals) of TG (**a**), HDL-cholesterol (**b**), LDL-cholesterol (**c**), and total-cholesterol (**d**) levels in participants treated with *C*. *annuum* compared with controls.

The forest plot for the overall effect of *C. annuum* supplementation on triacylglycerol (TG), which included results of three studies, is shown in Fig. 6. The pooled statistics showed that the SMD of TG was − 0.96 [95% CI − 2.67, 0.74; *P* = 0.27], which was not significantly different from that of the control group. The effect of *C. annuum* supplementation on TG showed considerable heterogeneity between studies [I^2^ = 95% and *P* < 0.01]. Sensitivity analyses of the effect of *C. annuum* supplementation on TG showed that removal of any study did not alter the significance of the pooled effect size but heterogeneity was lowered to 0% when the study of Cha et al.^24^ was removed (Supplementary Fig. S4). The forest plot for the overall effect of *C. annuum* supplementation on high density lipoprotein (HDL)-cholesterol, low density lipoprotein (LDL)-cholesterol, and total-cholesterol is shown in Fig. 6. *C. annuum* supplementation had no significant effects on HDL-cholesterol [SMD = 0.05; 95% CI − 0.28, 0.37; *P* = 0.78] and no significant between-study heterogeneity was noted in the analysis [I^2^ = 0% and *P* = 0.88]. Overall, four effect sizes from four studies were included in the analysis of the impact of *C. annuum* supplementation on total cholesterol and LDL-cholesterol. *C. annuum* supplementation had significant effects on LDL-cholesterol [SMD = − 0.39; 95% CI − 0.72, − 0.07; *P* = 0.02; prediction interval, − 1.28 to 0.50] compared to placebo supplementation. However, *C. annuum* supplementation had no significant effects on total-cholesterol [SMD = − 0.47; 95% CI − 1.06, 0.12; *P* = 0.12]. Statistical heterogeneity was detected in the analysis of total-cholesterol [I^2^ = 71% and *P* = 0.02] and LDL-cholesterol [I^2^ = 13% and *P* = 0.33]. Sensitivity analyses of the effect of *C. annuum* supplementation total-cholesterol showed that removal of any study did not alter the significance of the pooled effect size but lowered heterogeneity to 0% when the study of Cha et al.^24^ was removed (Supplementary Fig. S5).

### Adverse events

All RCTs for *C. annuum* supplements reported no serious adverse effects and no events leading to withdrawal. However, three studies from the intervention groups reported adverse events, including leg cramps^19^, dyspepsia^23^, bowel irregularities^23^, diarrhea^16,23^, skin rash^23^, heat sensation in the oral cavity^16^, and skin wheals^16^ , whereas there were no adverse effects in the placebo groups. Six studies reported no adverse events during the study^18,20–22,25,27^, and three studies did not report any information regarding adverse events^17,24,26^.

## Discussion

The aim of the present study was to investigate the effects of *C. annuum* on the factors related to MetS by focusing on the results of RCTs. Globally, MetS is considered as the main risk factor for CVD and T2D^2^. Several researches have been performed to investigate strategies such as dietary modification, which have a beneficial role in the prevention of MetS. A wide variety of herbs are considered as complementary and alternative treatments for MetS because they have beneficial effects on MetS and fewer side effects than conventional drugs. A systematic review and meta-analysis were performed to show the effects of green tea, *Nigella sativa*, and *Irvingia gabonensis* on MetS^28,29^.

In the present study, we found that *C. annuum* has marginally significant effects on body weight [SMD = − 0.19; 95% CI − 0.40, 0.03; *P* = 0.09]. Capsaicin is known to promote negative energy balance by increasing satiety and suppressing hunger, reducing energy and fat intake, and inducing thermogenesis^30–32^. Capsinoids, including capsiate and dihydrocapsiate, are also known to exert beneficial effects on energy balance. Reinbach et al., reported that CH-19 sweet pepper (non-pungent) reduces energy intake during positive energy balance. Additionally, CH-19 sweet pepper is associated with increased oxygen consumption, diet-induced thermogenesis, and an activated sympathetic nervous system (SNS)^33,34^. Furthermore, a meta-analysis revealed that capsiate increases energy expenditure (EE), but capsaicin has no overall effect on EE; however, both capsaicin and capsiate enhance fat oxidation^35^.

The effects of *C. annuum* on weight management are due to the activation of transient receptor potential vanilloid type 1 (TRPV1) receptors. In a preclinical study, dose-dependent administration of a TRPV1 agonist, capsaicin, prevented adipogenesis in stimulated 3T3-L1-preadipocytes, and oral administration of capsaicin prevented obesity in males^36^. Non-pungent capsiate is also an exogenous agonist of TRPV1 receptors and is associated with improved body metabolism including glucose homeostasis and obesity^37^. TRPV1 activation leads to activation of the SNS^38^, and inhibition of food intake by SNS activation accounts for reduced body fat stores and weight loss^38^. Some herbs and their active compounds, including several piperine analogs from *Piper nigrum,* can also reduce weight through TRPV1-dependent mechanisms^39–41^.

To the best of our knowledge, only a few meta‐analysis studies have investigated the anti‐obesity effects of *C. annuum*. After ingestion of capsaicin or capsinoids, EE increases and the respiratory quotient decreases, causing elevated fat oxidation^42^. Additionally, Zsiborás et al. showed that these metabolic effects of capsaicin and capsinoids are significant in individuals with a BMI greater than 25 kg/m^2^^42^. Another meta-analysis also indicated that both capsaicin- and capsiate-augmented EE enhance fat oxidation, especially in high doses. The findings of the meta-analysis by Whiting et al. suggest that reduced energy intake from daily consumption of capsaicinoids contributes to weight management^43^. Golzarand et al. in another meta-analysis reported a significant weight loss after consumption of capsaicin supplements (− 0.50 kg; 95% CI − 0.90 to − 0.11) but body fat percentage did not change significantly compared to baseline values (0.11%, 95% CI − 0.22 to 0.43, *P* = 0.51)^44^.

Unfortunately, we could not establish a significant effect of *C. annuum* on TG (*P* = 0.27) and HDL-cholesterol levels (*P* = 0.78), and total cholesterol (*P* = 0.12); however, LDL-cholesterol (*P* = 0.02) were affected by *C. annuum* supplementation. The China Health and Nutrition Survey showed that frequent consumption of spicy food items including chili pepper is associated with improved blood lipid profiles and decreased risk for CVD^45^. Further, a preclinical study indicated that red chili pepper ethanol extract exhibits hypolipidemic effects. This was demonstrated through decreased levels of total cholesterol, LDL-cholesterol, TG, and very low density lipoprotein, with simultaneous increased HDL-cholesterol levels in female albino Wistar rats undergoing high fat diets^46^. Dietary capsaicinoids reduced total plasma cholesterol, non-HDL cholesterol, and TG in hamsters undergoing a high-cholesterol diets, suggesting decreased cholesterol absorption^47^. Additionally, short-term supplementation of capsaicin decreases LDL-cholesterol and increases HDL-cholesterol in vivo^48^.

CVD, a leading cause of death, now accounts for approximately one third of all deaths globally^49^. According to the World Health Organization, most CVD cases can be prevented by ameliorating risk factors such as high levels of blood lipids. Our present study showed that *C. annuum* can help reduce the risk of CVD by decreasing LDL-cholesterol levels. The well-known anti-oxidant effect of peppers doubles these beneficial effects^50,51^. This is because increased oxidative stress has a crucial role in the development of CVD and dietary consumption of antioxidants has been associated with reduced risk of CVD^52,53^. Both anti-hyperlipidemic and anti-oxidant effects of *C. annuum* are expected to effectively reduce the risk of CVD.

As an agonist for TRPV1 receptors, it has been suggested that capsaicin or capsiate can improve glucose metabolism by decreasing inflammation, increasing adiponectin levels, and lowering glucose and insulin levels^37^. Although we did not observe any significant effects of *C. annuum* on blood glucose levels, a lot of studies reported the ameliorating effect of capsaicin or capsiate on glucose metabolism via various mechanisms including improved insulin sensitivity, increased insulin levels, and gut micro biota modulation in diabetic animal models^54–56^. Though *C. annuum* has been shown to have beneficial effects on the blood pressure of preclinical hypertensive rats^57^, we did not note any significant effects of *C. annuum* supplementation on DBP and SBP in meta-analysis of the present study. Therefore, well‐designed clinical trials are needed for further evidence on hypolipidemic, hypoglycemic, and hypotensive effects of *C. annuum,* which have been identified in previous preclinical studies.

The strengths of the present study include consideration of all clinical trials that investigated the health-related effects of *C. annuum* on the components of MetS including obesity, hypertension, hyperglycemia, and dyslipidemia. However, some limitations need to be considered. First, the effects of *C. annuum* on the components of MetS were investigated using a small number of included studies; thus, evidence to support the effects was not enough. We could not assess subgroup analysis, meta-analysis of variance or meta-regression, and publication bias. Second, the present meta-analysis included many studies with ROB. Third, there was significant between-study heterogeneity in the present study which may be explained by differences in the methods of *C. annuum* intervention, study design, study population, etc. Finally, we did not register this systematic review in PROSPERO, an international prospective register of systematic reviews. For future studies, registering systematic reviews in prospective registers such as PROSPERO is recommended to avoid unintended duplication and to ensure transparent research process.

In conclusion, the present study evaluated of the effects of *C. annuum* on factors related to MetS by focusing on the results of RCTs. Our results showed that *C. annuum* supplementation had significant effect on LDL-cholesterol and marginally significant effect on body weight. Larger and well-designed clinical trials are needed to investigate the efficacy and safety of this dietary supplement in the treatment of MetS.

## Methods

This systematic review and meta-analysis was reported in accordance with the Preferred Reporting Items for Systematic reviews and Meta-Analyses (PRISMA) guidelines^58^ (Supplementary Appendix 1).

### Search strategy

The databases of PubMed, Embase, Google scholar, and Cochrane Library were searched up to April 2020 to identify relevant published articles. We used the following search terms to identify relevant titles and abstracts: “*Capsicum annuum* OR chili pepper OR chilli OR pepper NOT black pepper”. Moreover, only the studies conducted in clinical settings and written in English were considered. Additional articles were identified via a manual search of the reference lists of related original articles, reviews, and meta-analyses. After removing duplicate results using the Endnote software, the titles and abstracts were screened by two authors (Hwan-Hee Jang and Young-Min Lee) using Rayyan QCRI. The relevant studies were reviewed for dual full-text screening. Disagreements were resolved through discussion.

### Inclusion criteria

The meta-analysis was performed using the ‘Patient/Population, Intervention, Comparison and Outcomes’ format to determine whether an intervention with *C. annuum* supplementation (I) compared with placebo supplementation (C) had any effect on the components of MetS (O) among participants (P). The outcomes of interest were as follows: obesity (BMI, body weight, and body fat), hypertension (SBP and DBP), hyperglycemia (blood glucose), and dyslipidemia (TG, total-cholesterol, LDL-cholesterol, and HDL-cholesterol). Parallel or crossover RCTs were included, and observational studies and review articles were excluded. Papers written in English and accessibility of the full-text publication were required for inclusion. Two authors independently reviewed data from all the studies that fulfilled the inclusion criteria and any conflicts was resolved by consensus.

### Data extraction and ROB assessment

Two reviewers extracted and recorded the following data from each included study: authors and year of publication, study design, study population (proportion of women, age, number of participants, and BMI), intervention (dose, type, and duration), the outcomes (BMI, body fat, blood glucose, blood pressure, blood cholesterol, etc.) (Table 1). The ROB assessment was independently conducted by two reviewers using the Cochrane ROB 2.0 tool for parallel RCTs^59^. This tool considers selection bias, performance bias, detection bias, attrition bias, and reporting bias. Any disagreements were resolved by consensus.

### Statistical analysis

We used SMD with 95% CI as effect size measures. If not reported^17–20,25,26^, the mean differences were calculated by subtracting the baseline mean from the post-intervention mean; the standard deviation (SD) differences were estimated using the following formula: SD_diff_ = √SD_B_^2^ + SD_F_^2^ − 2 × Corr × SD_B_ × SD_F_, where SD_B_ is the baseline SD and SD_F_ is the SD of the final measures in the study^60^. The correlation value was conservatively set at 0.5 to calculate the change in SD^61^. Due to clinical heterogeneity of the studies, including differences in study design, doses, and intervention, a meta-analysis of quantitative data was conducted using random-effects model. A forest plot was mapped to indicate the pooled SMD and the 95% CI. Between-study heterogeneity was tested by using forest plots visually. Thereafter, both the Q homogeneity test and I^2^ statistics were used to evaluate the statistical heterogeneity quantitatively. Generally, there is considerable heterogeneity when p value for Q statistics is less than 0.1 and I^2^ is more than 50%^62^. Sensitivity analysis was conducted to investigate the effect of each study on the pooled effect size. All analyses were performed using R Statistical Software version 4.0.2 (Foundation for Statistical Computing, Vienna, Austria, URL https://www.r-project.org/).

## Supplementary information

Supplementary Appendix.

Supplementary Figures.

## Data Availability

The datasets generated during and/or analyzed during the current study are available from the corresponding author on reasonable request.
