# Supplementary material for: Effects of Capsicum annuum supplementation on the components of metabolic syndrome: a systematic review and meta-analysis
Source: Sci Rep. 2020 Dec 1;10:20912. doi: 10.1038/s41598-020-77983-2 (PMC7708630; doi:10.1038/s41598-020-77983-2)
Supplement: Supplementary file 2 — Supplementary Figures. [file 41598_2020_77983_MOESM2_ESM.pdf]

**Supplementary Information**

# Effects of *Capsicum annuum* supplementation on the components of metabolic syndrome: A systematic review and meta-analysis

Hwan-Hee Jang, Jounghee Lee, Sung-Hyen Lee, Young-Min Lee

(a) Forest plot

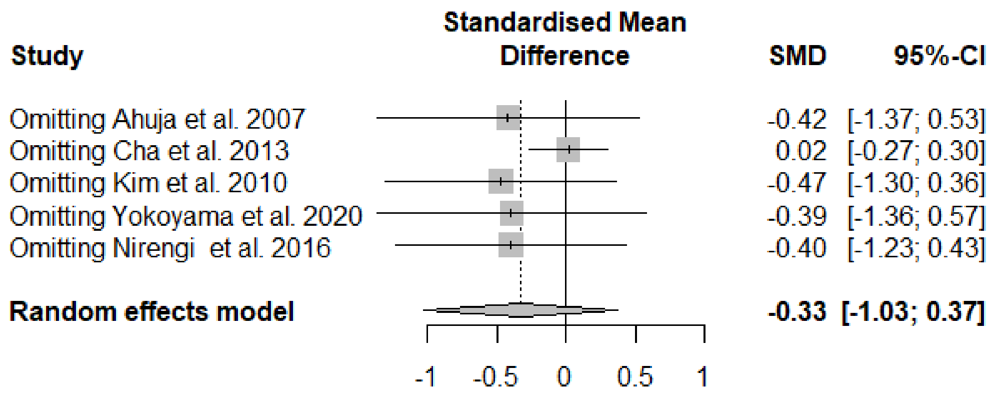


(b) Baujat plot

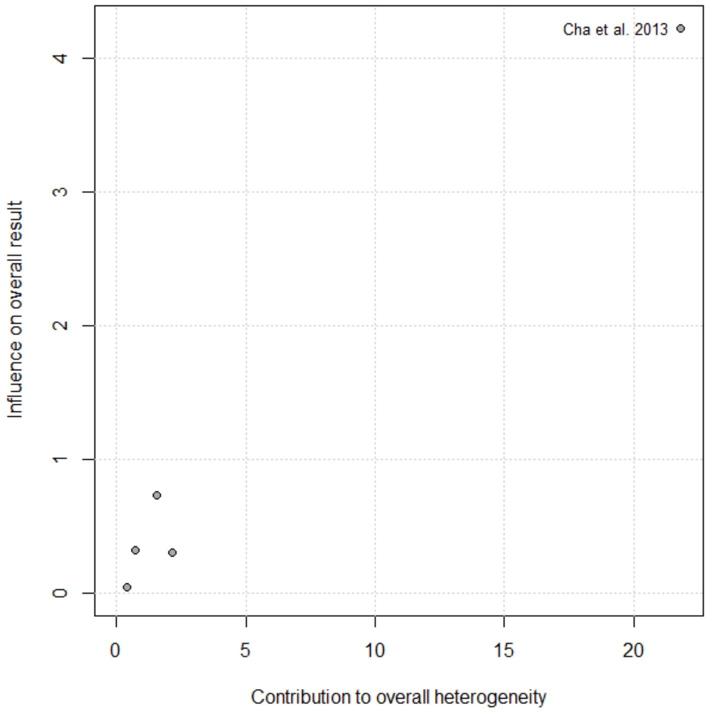


**Figure S1. Sensitivity analysis of individual studies showing the overall effect of *Capsicum annuum* supplementation on body mass index and the contribution to heterogeneity**

(a) Forest plot

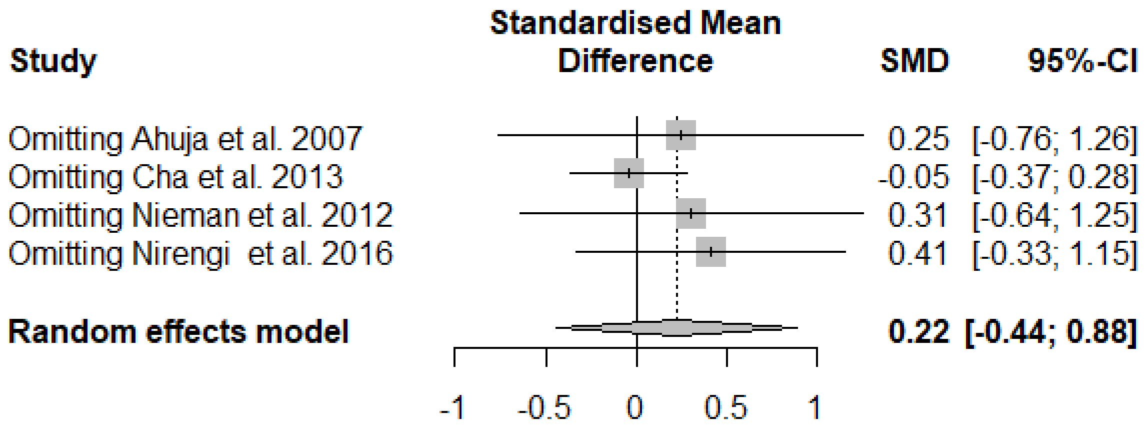


(b) Baujat plot

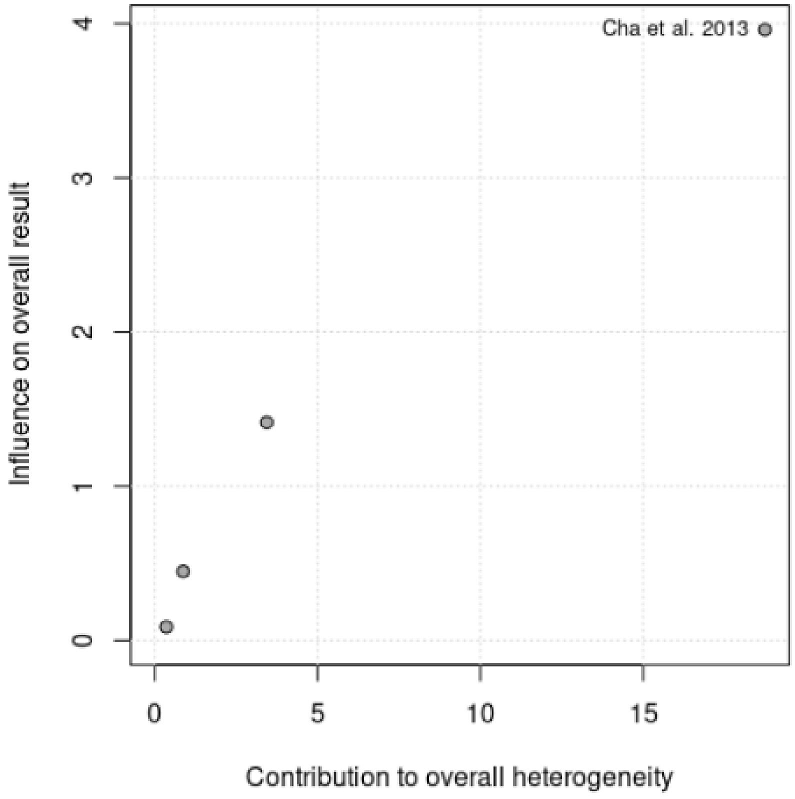


**Figure S2. Sensitivity analysis of individual studies showing the overall effect of *Capsicum annuum* supplementation on systolic blood pressure and the contribution to heterogeneity**

(a) Forest plot

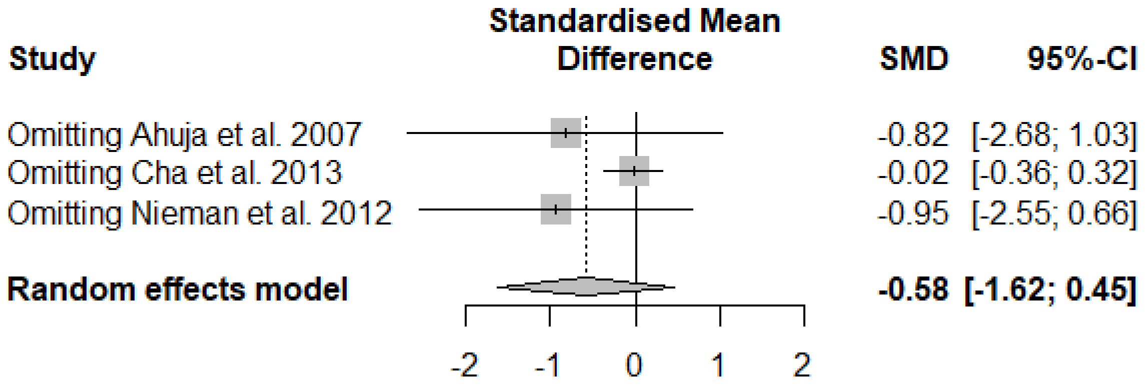


(b) Baujat plot

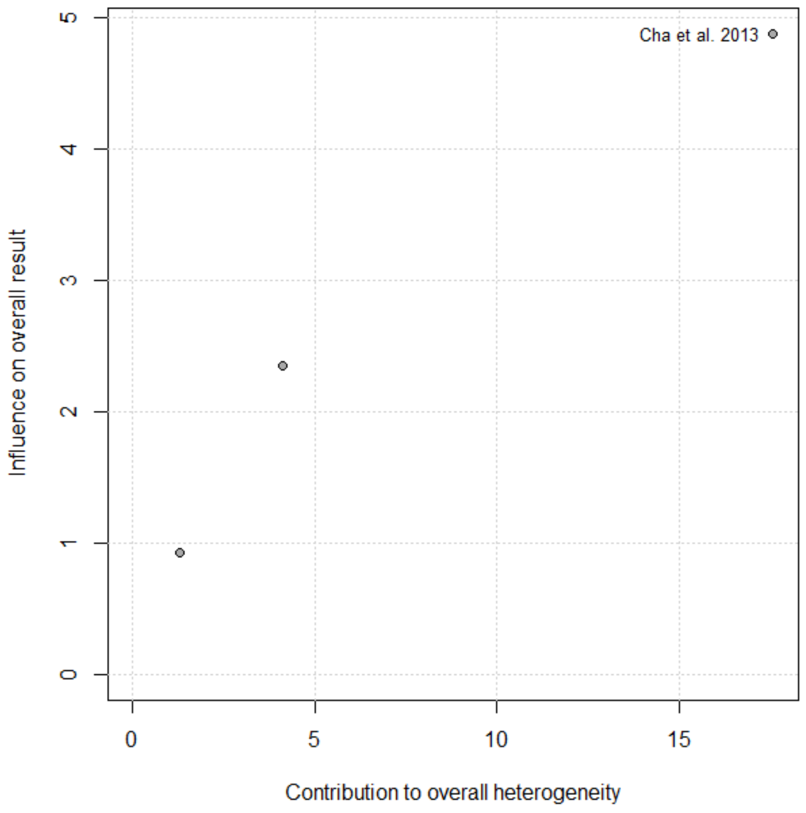


**Figure S3. Sensitivity analysis of individual studies showing the overall effect of *Capsicum annuum* supplementation on glucose and the contribution to heterogeneity**

(a) Forest plot

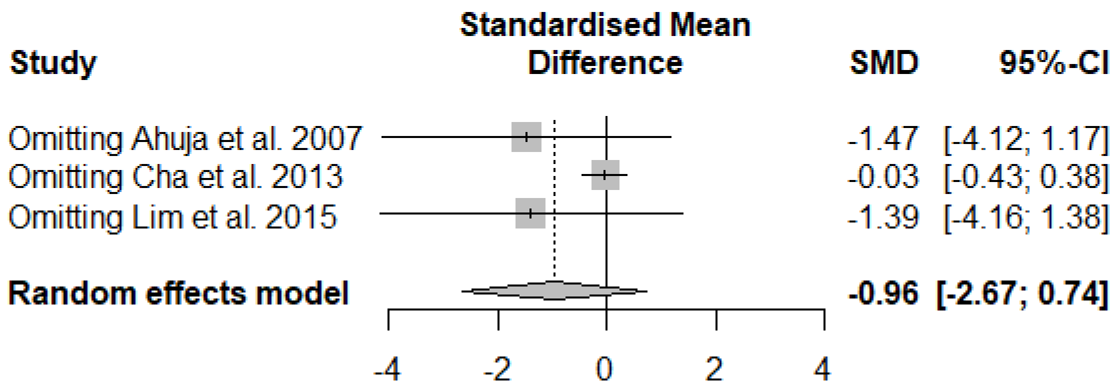


(b) Baujat plot

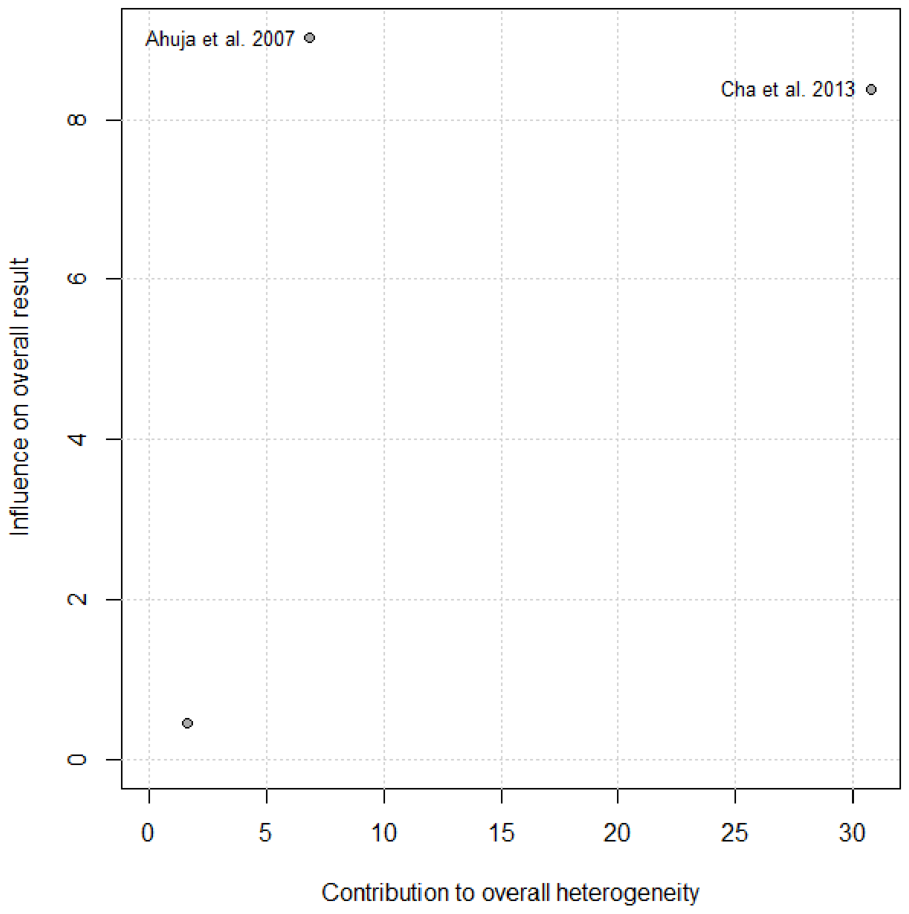


**Figure S4. Sensitivity analysis of individual studies showing the overall effect of *Capsicum annuum* supplementation on triacylglycerol results and the contribution to heterogeneity**

(a) Forest plot

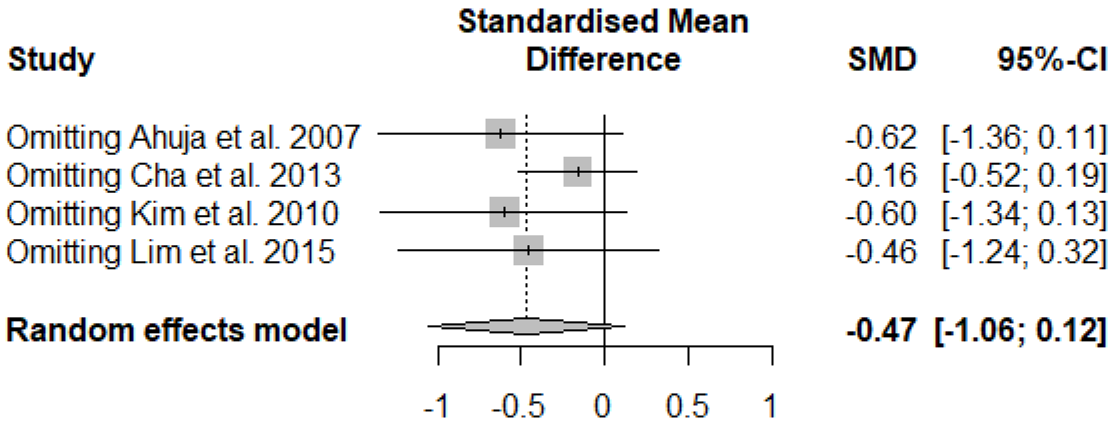


(b) Baujat plot

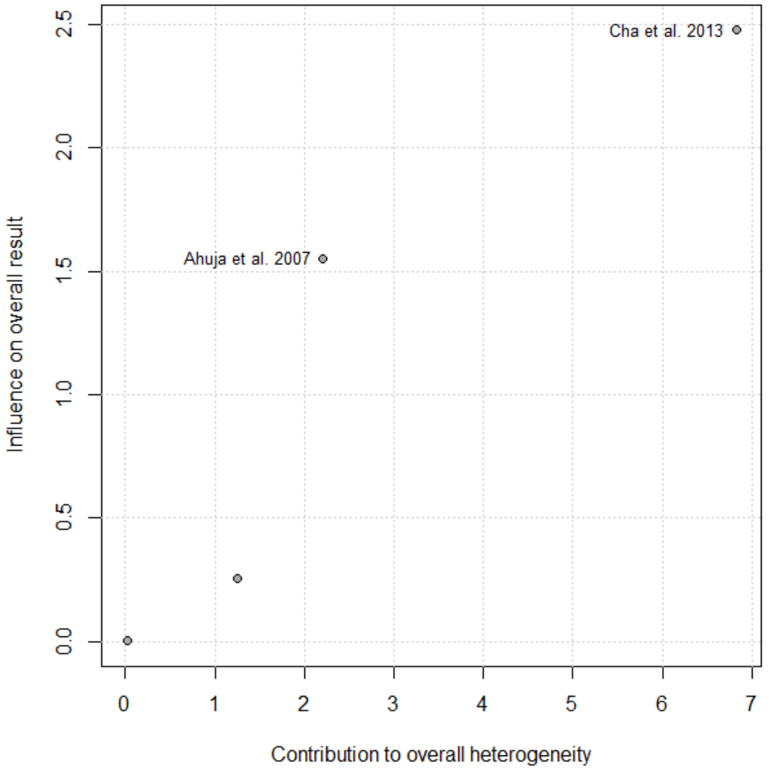


**Figure S5. Sensitivity analysis of individual studies showing the overall effect of *Capsicum annuum* supplementation on total cholesterol and the contribution to heterogeneity**
